# Female Reproductive Factors and Differentiated Thyroid Cancer

**DOI:** 10.3389/fendo.2017.00111

**Published:** 2017-05-23

**Authors:** Mariacarla Moleti, Giacomo Sturniolo, Maria Di Mauro, Marco Russo, Francesco Vermiglio

**Affiliations:** ^1^Unit of Endocrinology, Department of Clinical and Experimental Medicine, University of Messina, Messina, Italy

**Keywords:** thyroid cancer, pregnancy, reproductive factors, estrogens, estrogen receptors

## Abstract

Differentiated thyroid cancer (DTC) is markedly more common in women than men, the highest female-to-male ratio being recorded during the reproductive period. This evidence has led to the suggestion that female hormonal and reproductive factors may account for the observed DTC gender disparity. This review focuses on current evidence on the risk of DTC in conjunction with major female reproductive factors, including the impact of pregnancy on DTC occurrence and progression/recurrence. Overall, studies exploring the link between the risk of DTC and menstrual and menopausal factors, oral contraceptives and/or hormone replacement therapy, showed these associations, if any, to be generally weak. Nonetheless, there is some evidence that higher levels of exposure to estrogens during reproductive years may confer an increased risk of DTC. As far as pregnancy is concerned, it is unclear whether a potential association between parity and risk of DTC actually exists, and whether it is enhanced in the short-term following delivery. A possible role for pregnancy-related factors in DTC progression has been recently suggested by some reports, the results of which are consistent with a worse outcome in the short-term of women diagnosed with DTC during gestation compared to non-pregnant control patients. Also, some progression of disease has been described in women with structural evidence of disease prior to pregnancy. However, there seems to be no impact from pregnancy in DTC-related death or overall survival. Several *in vitro* and animal studies have evaluated the influence of estrogens (E) and estrogen receptors (ERs) on thyroid cell proliferation. Presently available data are indicative of a role of E and ERs in thyroid cancer growth, although considerable discrepancies in respect to ER expression patterns in thyroid cancer tissues actually exist. Further studies providing more direct evidence on the possible role of E and of placental hormones and growth factors on thyroid growth may expand our knowledge on the mechanisms beyond the gender disparity of proliferative thyroid diseases.

## Introduction

Thyroid cancer is the most common endocrine malignancy and the eighth most common cancer in the USA (1). With few exceptions, over the last decades a trend toward an increasing incidence of thyroid cancer has been described in several geographic areas (2–7). This raise has been attributed to the increased incidence of small papillary thyroid cancer (PTC), although an increase in follicular thyroid cancer incidence rates (IRs) has also been recorded in both sexes (8).

Overall, increased incidences of thyroid cancer in females are observed across different countries and ethnicities (3). Based on the National Cancer Institute’s Surveillance, Epidemiology, and End Results cancer statistics, the age-adjusted IR of thyroid cancer in 2013 was 21.61/100,000 women and 7.26/100,000 men, for a female-to-male (F/M) ratio of almost 3:1. However, because of differences between sexes in thyroid cancer peak age incidence, the F/M ratio is higher during the reproductive period (4.1:1 at ages 20–49 years), and steady decreases with advancing age (1.38:1 at ages ≥75 years) (http://seer.cancer.gov/faststats, accessed on December 2016). In addition, reports analyzing gender distribution according to the histotypes collectively reveal a twofold to almost fourfold F/M IR ratios for differentiated thyroid cancer (DTC), but a lack of gender disparity for the medullary and the anaplastic types (5, 9–11).

Both the preponderance of DTC incidence in females and the incidence peak in women of childbearing age suggest that hormonal and reproductive factors may account for the observed DTC gender disparity. Here, we briefly review current evidence on the role of the major female reproductive factors associated with DTC risk and the impact of pregnancy on thyroid cancer occurrence, progression, or recurrence.

## Methods

The terms *thyroid cancer*/*carcinoma* or *differentiated thyroid cancer/carcinoma* were used in conjunction with the terms *reproductive/menstrual/hormonal factors, reproduction, pregnancy* to search MEDLINE for articles published in English in the last 20 years (1996–2016). Additional papers were searched by scrutinizing the reference lists of previously published reviews and meta-analyses.

## Results

### Menstrual Cycling

Since the incidence of DTC in females abruptly increases at the time of puberty and declines after menopause (12), the relationship between menstrual factors and DTC risk has been addressed in a number of studies (13–31), the results of which are quite heterogeneous. Concerning age at menarche and DTC risk, most studies reported no significant associations, whereas other showed either early (<12 years) (16, 20, 29) or late (>15–16 years) (13, 16, 18, 27) age at menarche to be weakly associated with increased DTC risk. A history of irregular menstrual cycles has been reported to confer a higher risk of DTC, the magnitude of the observed associations being generally weak and/or limited to specific subsets of population (18, 27).

As far as menopause is concerned, most studies that examined the association of DTC with menopausal status, age at menopause, and type of menopause, failed to find consistent associations (14, 16–18, 21–25, 27, 28, 31). More recently, two meta-analyses showed postmenopausal status and older age at menopause to be, respectively, associated with a borderline significant reduction (RR 0.79, 95% CI 0.62–1.01) (32) and increase (RR 1.24, 95% CI 1.00–1.53) (33) of DTC risk. A further meta-analysis focusing on PTC only, also found late age at menopause to be associated with increased PTC risk (RR = 1.39, 95% CI 1.03–1.89) (34).

A roughly twofold increased DTC risk was reported among women who underwent surgical menopause compared to premenopausal women (13, 14, 19, 31). In a pooled analysis of 14 case–control studies, Negri et al. found that the OR was 1.8 (95% CI 1.4–2.4) for women with artificial menopause, compared to premenopausal women. The increased risk of DTC in women who underwent surgical menopause could be explained by an enhanced medical surveillance because of symptoms of sudden ovarian failure (35). Alternatively, since leiomyomas are the leading indication for hysterectomy globally and this condition is known to be associated with hyperestrogenism (36), the increased DTC risk associated with artificial menopause could result from the exposure to an underlying clinical condition—namely, hyperestrogenism—possibly favoring the development of both uterine myomas and thyroid cancer (30, 35). A role for hyperestrogenism *per se* is also supported by the findings from a prospective study consisting of naturally postmenopausal women only in which a greater length of exposure to endogenous sex steroid hormones during reproductive years was associated with an increased risk of DTC. Indeed, thyroid cancer risk was found to be decreased with increasing age at menarche [≥14 vs <12 years: hazard ratio (HR) 0.62, 95% CI 0.36–1.05] but increased with both increasing age at natural menopause (≥55 vs <50 years: HR 2.24, 95% CI 1.20–4.18) and greater lifetime number of ovulatory cycles (≥490 vs <415 cycles: HR 2.40, 95% CI: 1.33–4.30). Notably, in this study, a history of uterine leiomyomas was also associated with an increased risk of thyroid cancer (HR 1.72, 95% CI: 1.18–2.50) (30).

In summary, studies exploring the risk of DTC in conjunction with menstrual and menopausal factors yielded rather equivocal results, as the associations, if any, are generally weak. Nonetheless, there is some evidence that higher levels of exposure to estrogens during reproductive years may confer an increased risk of thyroid cancer.

### Use of Exogenous Hormones

Several large studies investigated the potential relation between oral contraceptive (OC) use and/or hormone replacement therapy (HRT) and DTC risk.

An analysis of pooled data based on 2,132 female thyroid cancer cases and 3,301 controls showed neither association between ever OC use and DTC risk nor relationship with duration or age at first use (37). However, the OR was higher for current OC users (OR 1.5, 95% CI 1.0–2.1), declining 10 years after stopping (OR to 1.1, 95% CI 0.8–1.4). Consistent with the above analysis, the vast majority of studies published afterward also showed ever use of OCs or age at first/last use to be generally unrelated to DTC, with no significant trends with increasing duration of use (14, 17, 18, 21–23, 25, 27, 28, 30, 38). In contrast, results from other studies suggest a reduced risk of DTC in OC users (16, 20, 31), either irrespective of duration (16, 20) or related to prolonged OC use only (26, 31). Interestingly, a reduced risk of DTC (HR 0.63, 95% CI 0.38–1.04) with use of *any* contraceptive method and with intrauterine devices use was observed in one study (39). This finding raises the question of whether the reduced DTC risk in OC users may be related to the lack of physiological thyroid changes during gestation (40) because of the prevented pregnancies rather than to a protective role of OC *per se*.

With few exceptions (26, 27, 31), studies providing information on HRT in relation to DTC overall indicate the use of estrogens in women of post-reproductive age not to modify cancer risk (13, 14, 16–18, 21, 22, 24, 25, 28, 30, 41, 42). A recent prospective study (31) showed that, after stratifying for HRT type, no association could be found with progestin alone or progestin and estrogen combined, whereas estrogen use alone was significantly associated with increased risk of DTC (HR 1.67, 95% CI 1.08–2.59).

The relationship between fertility-enhancing drugs and thyroid cancer risk has been less extensively investigated. In a pooled analysis, an overall OR of 1.6 (95% CI 0.9–2.9) was calculated on the basis of 22/730 women affected with DTC and 29/1,167 controls who had used fertility drugs (37). In a series of 8,422 women evaluated for infertility, 18 of whom diagnosed with DTC, neither clomiphene nor gonadotropin use significantly increased the risk of thyroid cancer (43). In another study by the same group, the above association was investigated on a larger population (9,892 women) overall including 55 thyroid cancer cases, and with a follow-up extending to an average of 30 years. Once again, no association with clomiphene use was observed, although DTC risk was significantly enhanced among the heavily exposed patients (HR = 1.96, for those receiving >2,250 mg) (44). By contrast, examining a cohort of 54,362 women with fertility problems, Hannibal found that women who had ever used clomiphene had a higher risk of thyroid cancer compared with those who had never used it (RRs 2.28, 95% CI 1.08–4.82), with the risk being primarily increased among parous women (RR 3.76, 95% CI 1.48–9.58) (44).

Overall, other fertility-enhancing drugs (follicle-stimulating hormone, human menopausal gonadotropin, gonadotropin-releasing hormone, or human chorionic gonadotropin) generally proved unrelated to DTC risk, with no association for all groups of fertility drugs relative to number of cycles or years since first use (43–47).

To summarize, current evidence does not support any strong relation between OC and/or HRT and DTC risk. Conversely, findings from some studies are suggestive of a potential association between clomiphene use and thyroid cancer. Mechanisms possibly responsible for the increased risk of DTC in clomiphene-exposed women are not clear, although a role for hyperestrogenism arising from ovarian stimulation has been postulated (44). Under such circumstances, estrogens, either directly (12) or indirectly, through enhanced serum thyrotropin (TSH) levels (48) may enhance mitotic activity in the follicular cells of the thyroid gland and promote thyroid cancer development/progression (49). However, if that is the case, the reasons for a lack of association between DTC and other ovulation-stimulating agents, equally effective in inducing a state of hyperestrogenism, remain elusive.

Table 1 summarizes the main findings from original studies reporting significant associations between menstrual factors and use of exogenous hormones and DTC risk.

**Table 1:** **Summary results of original studies reporting significant associations between menstrual factors, exogenous hormones, and thyroid cancer**.

Reference	Country	Study design	Study population (*n*)	Study population age (years)	DTC risk
Galanti et al. (13)	Norway, Sweden	Case–control	191 cases vs 341 controls	18–75	↑ For onset of menarche >15 years and for menopause >48 years of age
Mack et al. (14)	California (USA)	Case–control	292 cases vs 292 controls	15–54	↑ In women who had never menstruated regularly and in those who underwent bilateral oophorectomy
Rossing et al. (15)	Washington State (USA)	Case–control	410 cases vs 574 controls	18–64	↑ In women ≥45 years with surgical menopause (regardless of oophorectomy)
Sakoda and Horn-Ross (16)	California (USA)	Case–control	544 cases vs 558 controls	20–74	↑ For onset of menarche <12 or >14 years, with significant differences among age- and ethnic-specific subgroups ↓ In ever OC users (irrespective of duration)
Truong et al. (18)	New Caledonia	Case–control	293 cases vs 354 controls	>18	↑ For onset of menarche >15 years and in women who had never menstruated regularly, with significant differences among ethnic-specific subgroups
Brindel et al. (19)	French Polynesia	Case–control	201 cases vs 324 controls	<56	↑ In women with natural or surgical menopause
Xhaard et al. (20)	France	Case–control	633 cases vs 677 controls	<35	↑ For onset of menarche <12 years ↓ In ever OC users (irrespective of duration)
Schonfeld et al. (26)	USA	Cohort	312 cases from a cohort of 187,865 women	50–71	↓ In OC users (prolonged use only)
Horn-Ross et al. (27)	California (USA)	Cohort	233 cases from a cohort of 117,646 women	<80	↑ (in women age <45 years at baseline) for onset of menarche ≥14 years, longer (>30 days) adolescent menstrual cycles
Sungwalee et al. (29)	Thailand	Cohort	17 cases from a cohort of 10,767 women	30–69	↑ For onset of menarche <14
Braganza et al. (30)	USA	Cohort	127 cases from a cohort of 70,047 women	50–78	↑ For older age (≥55 years) at natural menopause and greater estimated lifetime number of ovulatory cycles
Zamora-Ros et al. (31)	Europe	Cohort	537 cases from a cohort of 345,157 women	Mean age 51	↑ In women with surgical menopause ↓ In OC users (prolonged use only)
Hannibal et al. (44)	Denmark	Cohort	29 cases from a cohort of 54,362 women	18–55	↑in clomiphene-treated women
Calderon-Margalit et al. (45)	Israel	Cohort	71 cases from a cohort of 15,030 women	Mean age ~28	↑ In clomiphene-treated women

*DTC, differentiated thyroid cancer; OC, oral contraceptive; ↑, increased risk; ↓, decreased risk*.

### Reproductive History

After breast cancer, DTC represents the second most common malignancy diagnosed during pregnancy and the early postpartum period in the USA (50).

In a case–control study nested within a cohort of Swedish women, a weak association between parity and risk of thyroid cancer (OR for ever-parous women vs nulliparous 1.1, 95% CI 1.0–1.3) was found. However, when the subset of PTC only was taken into account, there was a significantly increased risk for ever-parous vs nulliparous women (OR 1.3, 95% CI 1.0–1.6), along with a positive linear trend with increasing number of live births. In addition, women during the first year after a live birth had an increased risk of DTC compared with women who delivered 10 or more years before (51). Similarly, a population-based case–control study carried out in the USA showed a transiently increased risk of PTC among women who had delivered a live birth within the 5 years before the reference date, and the risk was even more pronounced among women with two or more births (OR 4.2, 95% CI 2.0–8.9) compared to women with one pregnancy only (OR 1.6, 95% CI 1.0–2.7) (15). A short-term effect of pregnancy on thyroid cancer risk is also supported by pooled estimates of large datasets (32, 34, 52, 53) and findings from other studies (16–18, 27, 31, 54). However, other investigations concluded that ever being pregnant, number of births and/or recency of pregnancies resulted in no increased (23, 26) or even decreased (22) risk of DTC (Table 2).

**Table 2:** **Summary results of original studies reporting significant associations between pregnancy and thyroid cancer risk/prognosis**.

Reference	Country	Study design	Study population (*n*)	Study population age (years)	DTC risk/prognosis
Rossing et al. (15)	Washington State (USA)	Case–control	410 cases vs 574 controls	18–64	↑ In women <45 years who had delivered one or more live births within the 5 years before DTC diagnosis
Sakoda and Horn-Ross (16)	California (USA)	Case–control	544 cases vs 558 controls	20–74	↑ In uni/multiparous women who had delivered one or more live births within the 5 years before DTC diagnosis
Truong et al. (18)	New Caledonia	Case–control	293 cases vs 354 controls	218	↑ In women <45 years with a trend toward an increase in risk with the number of full-term pregnancies
Navarro Silvera et al. (22)	Canada	Cohort	169 cases from a cohort of 89,797 women	40–59	↓ Among women with 5 or more live births vs nulliparous
Horn-Ross et al. (27)	California (USA)	Cohort	233 cases from a cohort of 117,646 women	<80	↑ In younger women who had delivered within the 5 years before DTC diagnosis
Zamora-Ros et al. (31)	Europe	Cohort	537 cases from a cohort of 345,157 women	Mean age 51	↑ In women who had delivered within the 5 years before DTC diagnosis
Galanti et al. (51)	Sweden	Case–control	1,409 cases vs 7,019 controls	15–59	↑ In uni/multiparous women, especially during the first year after a live birth vs women who delivered 10 or more years before
Vannucchi et al. (57)	Italy	Retrospective	DTC diagnosis: *n* = 47 ≥ 1 year after delivery *n* = 14 during pregnancy or <1 year after delivery *n* = 61 before pregnancy or nulliparous	<45	Poorer prognosis for DTC diagnosed during pregnancy or in the first year after delivery compared to tumors developed in non-gravidic periods
Messuti et al. (58)	Italy	Retrospective	DTC diagnosis: *n* = 152 ≥ 2 years after delivery *n* = 38 during pregnancy or <2 years after delivery *n* = 150 nulliparous	<45	Higher risk of persistence/recurrence of disease for DTC diagnosed during pregnancy or within the second year after delivery than in control groups

*DTC, differentiated thyroid cancer; ↑, increased risk; ↓, decreased risk*.

For all the above, it is unclear whether a potential association between parity and risk of DTC in women actually exists, and/or whether it is limited to the short-term following delivery. Possible explanations include a closer clinical surveillance in women who had recent pregnancies on one hand, and an actual promoting role for placental growth factors and hormones on the other. Indeed, there is evidence that childbirth is an increased risk factor for goiter and thyroid nodule growth (40, 55, 56), and a possible role for pregnancy-related factors in DTC progression has been postulated. In particular, compared to tumors developed in non-gravidic periods, DTC occurring during pregnancy or in the first-year postpartum was associated with a poorer prognosis (OR 15.88, 95% CI 4.01–62.77, *p* = 0.001) (57). Similarly, a retrospective analysis of 340 women affected with DTC and aged ≤45 years showed a significantly higher rate of persistent/recurrent disease among those diagnosed during pregnancy or within 2 years after delivery as compared to both nulliparous and women with DTC diagnosis >2 years after delivery (58).

Another issue concerns the potential adverse impact of pregnancy on the risk of disease progression in women diagnosed with DTC months to years prior to pregnancy. Studies comparing structural imaging and/or serum Tg levels before pregnancy and shortly after delivery in women previously treated for DTC overall indicate that pregnancy has no unfavorable impact in women free of disease prior to pregnancy (59–62) or in those with indeterminate or biochemical incomplete response (62). By contrast, some progression has been described in patients with structural evidence of disease prior to pregnancy (60–62). Yet, evidence is still insufficient to conclude that in this subset of women disease progression was related to pregnancy *per se* or it would have likewise occurred regardless of pregnancy.

Finally, two subsequent studies involving Japanese women affected with micro-PTC and selected for active surveillance without therapeutic intervention (63) came to opposite conclusions. In the first study (64), significant changes (≥3 mm) in the size of micro-PTCs were observed more frequently in women who had experienced at least one pregnancy during the follow-up period compared with an age-adjusted control group of non-pregnant female patients (44.4 vs 11.1%). However, a larger retrospective analysis including all patients who experienced pregnancy and delivery during the Hospital’s active surveillance program demonstrated the incidence of growth of micro-PTCs during pregnancy to be low at 8%, with no cases of lymph node metastases developing during pregnancy (65).

Thus, based on the few available retrospective studies the rates of disease recurrence/persistence are seemingly somewhat higher among women with DTC occurring during pregnancy than in non-pregnant control patients. Importantly, as far as long-term outcome is concerned, there seem to be no impact from pregnancy in DTC-related death or overall survival (66–69) Also, presently available data indicate that pregnancy does not impact thyroid cancer prognosis in patients with no evidence of persistent disease prior to pregnancy. Conversely, whether pregnancy actually poses any risks for progression in women with preexisting structural disease, and whether this event is related to or independent of pregnancy, needs to be further investigated.

### Estrogen and Estrogen Receptors (ERs) in Benign and Malignant Thyroid Diseases

The evidence of a three to four times higher prevalence of proliferative thyroid diseases in females than in males and their peak incidence in premenopausal women strongly suggests that female sex hormones may play a role in the pathogenesis of both benign and malignant thyroid tumors. Several *in vitro* and animal studies have evaluated the influence of sex steroids on proliferation of thyroid cells, with estrogens (E) and ERs being the most investigated target of current research.

Either endogenous or exogenous hyperestrogenism are known to indirectly affect thyroid function, through an increase of serum thyroxine binding globulin (TBG) concentrations, due to both a stimulation of liver TBG synthesis and a reduced plasma clearance of the protein (43). This event is particularly marked during pregnancy and is accompanied by a trend toward a reduction in free-thyroid hormone concentrations and a subsequent stimulation of the pituitary–thyroid axis. In condition of iodine sufficiency, the enhanced thyroidal stimulation during pregnancy has no or little impact on thyroid function and growth. In contrast, a less than adequate iodine supply throughout gestation results in sustained hyperstimulation of the maternal thyroid gland, which may be responsible for goiter occurrence and/or thyroid nodules enlargement (70). Notably, gestational goiter may only partially regress after parturition (71), which may partly explain the preponderance of goiter in the female population. In addition, several lines of evidence demonstrate that 17-β-estradiol (E2) stimulation induces cell growth in primary cultures of human thyrocytes from benign and malignant thyroid nodules, FRTL-5 rat thyroid cell line, and in most human thyroid carcinoma cell lines (72–76). Also, *in vitro* E2 exposure enhances metastatic properties of both normal and thyroid cancer cell lines by targeting the expression of molecules involved in cell adhesion, migration, and invasiveness, such as β-catenin, E-cadherin, vimentin, and matrix metalloproteinases (77–80). The growth-promoting effect of E2 on thyroid cells is mediated by a classical genomic pathway involving nuclear ERs and by rapid non-genomic signaling events *via* a membrane-associated estrogen receptor (12, 81). The latter involves both the mitogen-activated protein kinase and the phosphatidylinositol 3-kinase signaling pathways, which are known to play a key role in thyroid tumorigenesis.

The expression of ERs has been investigated by immunohistochemistry, binding assays, and RT-PCR in a number of studies, the results of which are quite heterogeneous, irrespective of the technique employed (10, 12, 82). Nevertheless, both ERα and ERβ have been detected in variable proportions in normal thyroid tissue, as well as in benign and malignant thyroid lesions and in most thyroid cancer cell lines (82). The expression of ER isoforms would be differentially regulated by E2, since E2 treatment of human thyroid papillary carcinoma cells (KAT5) was found to significantly increase the expression of ERα but did not affect that of ERβ (76). Present evidence indicates that ERα and ERβ exert opposite functions on thyroid cancer cell, with ERα being responsible for cell proliferation and ERβ having pro-apoptotic functions (81). Mechanisms beyond these effects involve changes in the balance between the antiapoptotic B-cell lymphoma 2 (Bcl-2) protein and the pro-apoptotic Bax protein, which are upregulated by ERα and ERβ, respectively (76). Interestingly, an increase in ERα but a decrease in ERβ expression has been observed in human thyroid cancer cells (83, 84), which suggests that, as for breast cancer, an imbalance in the expression of the two isoforms may favor human thyroid cancer progression (76, 81). Consistent with this hypothesis are the findings from some recent clinical studies, overall arguing for an association between ERα expression and/or partial or total lack of ERβ expression in DTCs and a more aggressive presentation or a trend toward the presence of local metastases at diagnosis (85–87). Relative to the expression of ERs in DTC diagnosed during pregnancy, one study found the vast majority (87.5%) of tumors diagnosed during or soon after pregnancy to test positively for ERα, which, according to the authors, could explain the worse outcome observed in this subset of patients compared to non-pregnant women (57). Another study, however, while confirming a significant correlation between pregnancy and a poorer outcome of DTC, found the immunohistochemical expression of ERα and ERβ to be globally low and unrelated to the time of tumor diagnosis in respect of pregnancy (58).

A role for E2 in thyroid cancer occurrence is also suggested by the higher than expected rates of thyroid cancer in women with a history of breast cancer and *vice versa*. Indeed, a recent meta-analysis focusing on this issue showed the ORs of developing thyroid or breast cancer as secondary malignancy after diagnosis with the other to be increased at 1.55 (95% CI 1.44–1.67) and 1.32 (95% CI 1.23–1.42), respectively (88).

Mechanisms underlying the bidirectional association between thyroid and breast cancer are still unclear and may involve, either separately or in combination, both exogenous (i.e., increase in surveillance, radiation exposure, environmental risk factors) and endogenous factors (88). The latter ones include genetic susceptibility, as is the case for Cowden and Cowden-like syndromes (89), as well as a common molecular mechanism mediated by sex steroid receptors. In this regard, compared to control groups of breast cancer as the only malignancy, the expression of both ER and progesterone receptor (PR) was found to be significantly increased in breast cancer specimens from women who also had DTC, regardless of which of the two cancers had occurred first and/or of the latency to detection of the second malignancy (90, 91). Interestingly, evidence has been provided showing that rearranged during transfection/papillary thyroid carcinoma (RET/PTC) oncogene is induced by estrogen in breast cancer cells, and the RET/PTC kinase signaling enhances estrogen-driven cell proliferation (92, 93). Since RET/PTC rearrangements are found in approximately 20% of spontaneous PTCs (94), a shared mechanism mediated by ER/PR signaling and involving the RET/PTC-dependent tyrosine kinase pathways might underlie the observed association of breast cancer and thyroid cancer.

## Concluding Remarks

Presently available clinical and experimental data are indicative of a role of estrogens and their receptors in thyroid cancer growth. Nonetheless, considerable discrepancies between studies in respect to ER expression patterns in thyroid cancer tissues actually exist. Also, it is still unclear whether the pregnancy-related hyperestrogenic state may account for the poorer outcome observed in women diagnosed with DTC during gestation. In this regard, it should be considered that a number of placental hormones (other than estrogens) and growth factors (i.e., insulin-like growth factors 1 and 2, epidermal growth factor, vascular endothelial growth factor, and members of the transforming growth factor-β superfamily) are increased within the maternal circulation throughout gestation (95) and might theoretically contribute to thyroid cancer occurrence/progression in pregnancy. Further studies providing more direct evidence for a role of E2 in thyroid carcinogenesis, and investigations on the effects, if any, of placental growth factors on thyroid growth, may be helpful in understanding the mechanisms beyond the gender disparity of proliferative thyroid diseases.

## Author Contributions

All the authors listed have made substantial, direct, and intellectual contribution to the work and approved it for publication.

## Conflict of Interest Statement

The authors declare that the research was conducted in the absence of any commercial or financial relationships that could be construed as a potential conflict of interest.
